# Cuisine in transition? Organic residue analysis of domestic containers from 9th-14th century Sicily

**DOI:** 10.1098/rsos.221305

**Published:** 2023-03-08

**Authors:** Jasmine Lundy, Lea Drieu, Paola Orecchioni, Antonino Meo, Veronica Aniceti, Girolamo Fiorentino, Milena Primavera, Helen Talbot, Alessandra Molinari, Martin O. H. Carver, Oliver E. Craig

**Affiliations:** ^1^ Department of Archaeology, BioArCh, University of York, York YO10 5ND, UK; ^2^ Université Côte d'Azur, CNRS, CEPAM, Nice UMR 7264, France; ^3^ Dipartimento di Storia, Patrimonio Culturale, Formazione e Società, Università degli Studi di Roma Tor Vergata, Rome 00133, Italy; ^4^ ’Antonino Sallinas’, Regional Archaeological Museum of Palermo, Palermo 90133, Italy; ^5^ Department of Natural History, University Museum of Bergen, Bergen 5007, Norway; ^6^ Laboratory of Archaeobotany and Palaeoecology, Università del Salento, Lecce 73100, Italy

**Keywords:** archaeology, medieval Sicily, organic residue analysis, cuisine, ceramics

## Abstract

From the 9th to 14th centuries AD, Sicily experienced a series of rapid and quite radical changes in political regime, but the impact of these regime changes on the lives of the people that experienced them remains largely elusive within the historical narrative. We use a multi-faceted lipid residue approach to give direct chemical evidence of the use of 248 everyday domestic ceramic containers from Islamic and post-Islamic contexts in western Sicily to aid our understanding of daily habits throughout this period of political change. A range of commodities was successfully identified, including animal fats, vegetable products, fruit products (potentially including wine) and plant resins. The study highlights the complexity of residues in early medieval Mediterranean society as, in many cases, mixtures of commodities were observed reflecting sequential cooking events and/or the complex mixtures reflective of medieval recipes. However, overall, there were no clear changes in the composition of the residues following the imposition of Norman control over the island and through subsequent periods, despite some differences between urban centres and rural sites. Thus, lending to the idea that post-Islamic populations largely flourished and benefited from the agricultural systems, resources and recipes left by their predecessors.

## 1. Introduction

Understanding the impact of major political changes in the past on everyday social and economic practices is a major goal in historical archaeology. The Mediterranean island of Sicily has become a focus point for these investigations, given its long history of colonization and control by different political ‘regimes’. The 9th-14th centuries were particularly tumultuous. During this period, the inhabitants of the island experienced socio-economic, political and religious change, with four successive regimes: Islamic, Norman, Swabian and Aragonese. However, the impact of these changes on the lives of farmers, merchants and their families was largely undocumented and therefore requires careful archaeological investigation to be understood [1]. By undertaking chemical analysis to determine the use of domestic cooking pots over this 600-year period, here we explore changes in regime on the population of Sicily through the lens of cuisine.

Cuisine, the way in which foods are combined, prepared and consumed, can yield important insight into everyday life. Culinary habits are often linked, but by no means limited to, socio-economic factors such as food availability and foodscapes, wealth, faith, and the technologies available for the procurement and processing of food. It is hypothesized that the complexity of the transitions experienced in Sicily from the 9th to 14th centuries are probably reflected in the culinary habits of the populations that experienced them. As utilitarian artefacts, domestic cooking pots that are routinely found through archaeological investigation might be expected to capture a significant subset of foodstuffs processed daily. Organic residue analysis (ORA) of these domestic cooking wares can give direct chemical evidence of their contents and yield important insight into the types of foodstuffs that were prepared and combined by the communities that used them.

A recent study of Islamic cooking pots and other domestic containers from 9th-12th century contexts in Sicily has shown that a wide range of animal products, fruits and vegetables were processed, and has yielded important insight into the use of resources in both urban and rural contexts under the same regimes [2]. Previously, residue analysis has been undertaken on pottery from Italian 12th-13th century contexts [3–7], and this approach has also been used to examine the impact of the Norman conquest of England on culinary habits, with particular focus on lesser represented rural populations [8]. However, to date, no study has assessed the use of pottery vessels in post-Islamic contexts in Sicily.

To further understand the impact of these regime changes on culinary choices, 114 cooking wares obtained from Islamic, Swabian and Aragonese contexts from the urban site of Mazara del Vallo and Norman and Swabian contexts from the rural settlement of Casale San Pietro were analysed using ORA. These results are compared with 134 previously published data from Islamic contexts from Palermo and Casale San Pietro [2] to enable an in-depth comparison of pottery use in western Sicily over this significant period of transition and between different socio-economic settings (total 248 samples).

In order to gain greater depth of understanding regarding the commodities used over this period, a multi-faceted organic residue approach was implemented. A range of gas chromatography (GC) and mass spectrometry techniques were applied to identify these residues including selected ion monitoring (SIM) modes to identify aquatic biomarkers and alkylresorcinols [9,10] and gas chromatography-combustion-isotope ratio mass spectrometry (GC-C-IRMS) [11] to help distinguish between animal fats [12,13] (see Methods). We also employed a further extraction method to extract small organic acids to support identification of grapevine and other fruit products in these ceramics [14–16].

The primary objectives were to address the following questions:

1. How do the contents of domestic containers from Norman, Swabian and Aragonese contexts compare with domestic containers from Islamic contexts in Sicily?
2. How were culinary practices changed or maintained in both urban and rural settings as a result of these transitions?

## 2. Historical context

Sicily was a major supplier of grain to both the Roman and Byzantine imperial capitals and was an entrepôt between these great cities and North Africa, helping to maintain the Mediterranean triad of wheat, wine and olive oil. During the 8th century AD, Sicilian trade diminished, and the people of Sicily were obliged to concentrate on feeding themselves. This was achieved in large farming settlements of which Casale San Pietro in central-west Sicily is an example [17]. Following a century of skirmishing, the Muslim army arrived at Mazara in 827 AD and in 831 AD conquered Palermo to set up its capital there. The eventual conquest of the island from the Byzantines in the early 10th century and especially the assumption of power by the Kalbid dynasty led to a period of considerable prosperity. It reopened the door to imports from North Africa and from other parts of the Fatimid empire, notably the new fruits, vegetables and fibre crops, such as rice, sorghum, sugar cane, cotton, spinach, artichokes and citrus fruits, predicted in Watson's 1974 study and endorsed here by botanical evidence from Islamic Mazara [18]. This enlarged the diet, as did the introduction or breeding of larger specimens of sheep [19]. At the same time, chemical analysis of amphorae has shown that the Islamic regime began exporting wine to Christian and Islamic ports alike [14].

The Norman conquest of 1061 did not immediately extinguish Islamic culture, which maintained an important profile in culture, learning and language until the death of Roger II, and his successors at the end of the 12th century. In the 13th century, Sicily fell under the rule of the Swabian and eventual Holy Roman Emperor Frederick II (1198–1250). Muslims then came under considerable pressure culminating in mass deportations to mainland Italy in 1246. In Norman and Swabian times, there may have been a move back to cereals, vines and olives [20]. While the expectation might be that medieval political control would determine aspects of culture, our broader aim here is to uncover the actual life patterns of the Sicilian people, aspects of which might have followed a very different trajectory. Diet and cuisine are particularly strong indicators of cultural allegiance and are the focus of our study that uses the contents of domestic cooking pots as a proxy.

## 3. Sites and samples

### 3.1. Mazara del Vallo

**Figure 1.**
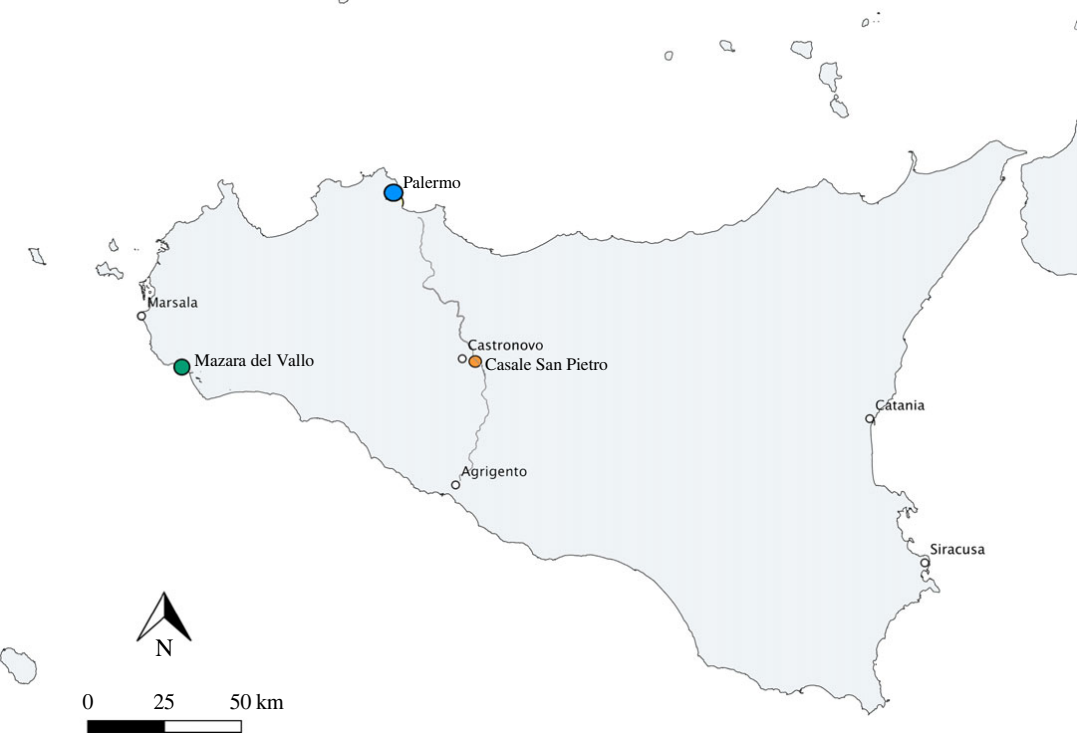
Map of Sicily showing the location of Mazara del Vallo, Casale San Pietro and Palermo. The main road between Palermo and Agrigento from north to south is marked on the map.

Mazara del Vallo (MZ) (Trapani) is located on the southwestern coast of Sicily (figure 1). Archaeological investigations in the northwestern part of the town, performed in 1997, have revealed streets with domestic residences and cesspits that serve them. Excavations also reveal a limestone surface with buildings, silos, wells and ditches that cut the ground, dated from the late 7th/ early 8th to the late 17th-18th centuries [21]. Based on these finds, the first Islamic phase was dated to the late 10th-11th century by pit latrines. Later, during the 11th century, after the demise of the major part of the latrines, a pottery workshop was built with pits and a furnace. After the demolition of the pottery workshop, life continued at Mazara del Vallo until the modern period. The majority of 11th century ceramics analysed in this study were obtained from the usage and abandonment layers of a waste pit that served the domestic residences of the town (Latrine 5) from US (19, 27, 31, 45 and 55) [22]. The radiocarbon date from a chicken from US 19 dates between 900-1030 AD at 95% confidence [23]. Two 11th century ceramic samples analysed in this study were obtained from the abandonment layers of the pottery workshop (US 78). Although Palermo was a major ceramic production site during the 9th-11th centuries, Mazara did not rely on kitchen wares produced in Palermo at this time, unlike other places such as Casale San Pietro [2,24]. Instead, Mazara produced its own local ceramics, but because of its coastal location it also received imports from elsewhere, particularly from North Africa. This depicts strong links between Mazara and North Africa at this time. The vessel types from this assemblage consist of cooking pots, pans, stone plates, braziers/tripods and some jugs with evidence of soot marks (electronic supplementary material, S2).

The later pottery comes from decommission of the wells belonging to the mid-13th century (Well 1: US 7), the late 13th century (Well 3: US 52, 71) and the mid-14th century (Well 4: US 176). In these contexts, there is a shift in the types of containers. Of note, there is a rise in glazed cooking wares, particularly partially glazed wares from Messina, where the rim and internal base of the vessel were glazed [25,26]. From 13th-14th century contexts, a selection of partially glazed cooking pots, unglazed pots, bowls/pans, *pentolino* (small cooking pots) and a jug were selected for analysis (electronic supplementary material, S2). The samples selected for this study are from contexts associated with the use of domestic residences and have been dated based on their typology and associated radiocarbon dates [26].

### 3.2. Casale San Pietro

Casale San Pietro (CLESP) is a site located on the plain of the river Platani outside of the town of Castronovo di Sicilia, in the rural hinterlands of the Palermo province. Excavations in 2016–2019 revealed that the site flourished during the 10th-11th centuries [24,27,28]. An impressive assemblage of ceramics, faunal remains and other material culture was recovered from this period and is described in full elsewhere [2]. In the 12th century, the site was still occupied, either as a small agricultural settlement or a station on the main road between Agrigento and Palermo, and evidence suggests, at least in the early 12th century, new buildings were built, using the pre-existing structures of the Islamic and Roman phases. Towards the later 12th century into the 13th century, it is likely that inhabitants predominantly occupied the nearby town of Castronovo and the Norman castle of San Vitale on the hills overlooking Casale San Pietro. However, ceramics dated to the 12th and 13th centuries found at Casale San Pietro suggests that there was still some domestic occupation at the site in the Norman and Swabian periods, but with a reduced economic profile [24,27,28]. The 10th-12th century ceramics previously studied by ORA consisted of *ollae*, cooking pots, pans and stone plates. In contrast with Mazara, these ceramics were predominantly imported from Palermo, with a small number of handmade pottery and calcite wares of unknown provenance [2]. In the 12th century, trade networks of ceramics into CLESP changed and new types were observed [24,27,28]. In this assemblage, we see the introduction of new glazed wares imported from northeast Sicily like those in Mazara. In the 13th century, perhaps unsurprisingly, there is a lesser variety in these cooking wares in comparison with the urban centre of Mazara [24,27,28]. Nonetheless, a selection of cooking pots and pans, glazed, partially glazed and unglazed, were analysed in this study (electronic supplementary material, S1).

### 3.3. Palermo

The ORA of these ceramics was compared with previously published ORA results from 9th-11th century ceramics from three sites in the urban capital of Palermo. The details of these sites and the ceramics studied are described in full elsewhere [2], but, in brief, a total of 83 vessels were obtained from Castello San Pietro (CSP), La Gancia (GA) and Palazzo Bonagia (PB). These consisted of *ollae*, cooking pots, braziers, lids, pans and stone plates [2,29–31].

## 4. Methods

### 4.1. Lipid extraction

Following common protocol for the extraction of absorbed residues [2], each ceramic sample was first ‘cleaned’ by removing the outer layer of the ceramic surface before collecting approximately 2 g of ceramic powder by drilling into the pot. An acidified methanol extraction (AE) method was applied to all samples following common procedure [32]. In brief, a mixture of methanol (4 ml) and sulphuric acid (800 µl) was added to 1 g of ceramic powder alongside an internal standard (10 µl of C_34:0_) before heating at 70°C for 4 h. After centrifuging, the supernatant was extracted and transferred to a clean labelled hatch tube. The lipid extract was separated from the acid by adding 2 ml of *n*-hexane, removing the supernatant and passing through a filter pipette to neutralize the sample with potassium carbonate (K_2_CO_3_). This process was repeated three times. The samples were dried under a gentle stream of nitrogen, and then dissolved in *n*-hexane and transferred to an auto-sampling vial with a micro insert. A second internal standard (10 µl of C_36:0_) was added before analysis by GC techniques.

A solvent extraction (SE) was undertaken on the majority of samples to extract and identify tri-, di- and mono-acylglycerols and/or wax esters, which do not remain intact after AE. Briefly, a mixture of dichloromethane-methanol (DCM 2 : 1, v/v) was added to the remaining 1 g of ceramic sample. The samples were sonicated for 15 min and then centrifuged. The supernatant was transferred to a clean labelled hatch tube. These steps were performed three times. The solution was then dried to completion under a gentle stream of nitrogen.

The ceramic powder remaining after SE was then submitted to acidified butanol extraction (ABE) [15,16]. Here, 2 ml of *n*-hexane and 1 ml of boron trifluoride solution (BF_3_) in butanol were added to the sample. After heating for 2 h at 80°C, the samples were centrifuged. The supernatant was then transferred to a clean labelled hatch tube and neutralized using a saturated solution of sodium carbonate. To ensure no more sample was trapped in the ceramic powder, 2 ml of DCM was added to the sample, vortexed, centrifuged and the supernatant transferred to the same extract. After centrifugation, the organic phase was transferred to a new clean labelled hatch tube. The aqueous phase was extracted twice more with DCM. The combined organic phases were washed twice with water, and dried under nitrogen. The dried extracts from SE and ABE were derivatized by adding *N,O*-bis(trimethylsilyl)trifluoroacetamide (BSTFA) with 1% trimethyl-chlorosilane and heated for 1 h at 70°C. *n*-hexane was added to redissolve the extract before transferring to an auto-sampling vial which contained 10 µl of the second internal standard C_36:0_ for GC analysis.

### 4.2. Gas chromatography-mass spectrometry

AEs and SEs were first screened using GC fitted with a flame ionization detector (FID) for quantification and general screening of preservation. An Agilent 7890A Series gas chromatograph was fitted with a DB1-high temperature (HT) column (15 m × 0.32 mm × 0.1 µm). One microlitre of the extract was injected via a splitless injector maintained at a temperature of 300°C. The temperature of the column was kept at 100°C for 2 min and then increased by 20°C every minute until a final temperature of 325°C was reached. A temperature of 325°C was then held for 2 min. Helium was used as the carrier gas at constant flow. The detector was kept at 300°C with hydrogen flow of 30 ml min^−1^. For SEs, the temperature of the column was kept at 50°C for 2 min and then increased by 10°C every minute until a final temperature of 375°C was reached. A temperature of 375°C was then held for 10 min.

To identify molecular profiles, all extracts were analysed using GC-MS. For analysis of AEs and ABEs, the GC component was an Agilent 7890A series attached to an MS Agilent 5975 Inert XL mass selective detector with a quadrupole mass analyser (Agilent Technologies, Cheadle, UK). A DB-5MS (5%-phenyl)-methylpolysiloxane column (30 m × 0.250 mm × 0.25 µm; J&W Scientific, Folsom, CA, USA) was used. The GC column was inserted directly into the ion source of the mass spectrometer. One microlitre of sample was injected via a splitless injector maintained at a temperature of 300°C. Helium at constant flow was used as the carrier gas. The ionization energy of the spectrometer was 70 eV and spectra were obtained by scanning between *m/z* 50 and 800. The temperature of the column was kept at 50°C for 2 min and then increased by 10°C every minute until a final temperature of 325°C was reached. A temperature of 325°C was then held for 15 min. For SEs, a HT column and programme were used to detect the presence of tri-, di and mono-acylglycerols (TAGs, DAGs and MAGs) and wax esters. A DB5-HT column (30 m × 0.25 mm × 0.1 µm) was used, and the temperature of the column was kept at 50°C for 2 min and then increased by 10°C every minute until a final temperature of 375°C was reached. To target ions specific to alkylresorcinols SEs were analysed using the same chromatographic conditions with the mass spectrometer in SIM mode. The ions *m/z* 73, 268, 464, 492, 520, 548, 576, 604 and 632, corresponding to alkylresorcinols with cyclic carbon chain lengths C_17_ to C_25_, were monitored.

AEs were also analysed using an Agilent 7890A series chromatograph attached to an MS Agilent 5975 Inert XL mass selective detector with a quadrupole mass analyser (Agilent Technologies, Cheadle, UK) equipped with a DB-23 (50%-Cyanopropyl)-methylpolysiloxane column (60 m × 0.250 mm × 0.25 µm; J&W Scientific, Folsom, CA, USA). The temperature of the column was kept at 50°C for 2 min and then increased by 10°C every minute until 100°C. The temperature increased then until 140°C by 4°C every minute, then until 160°C by 0.5°C every minute and finally until 250°C by 20°C every minute. A SIM mode was used to target different groups of ions. These groups were: *m/z* 74, 105, 262, 290, 318 and 346 for the detection of ω-(o-alkyl phenyl)alkanoic acids of carbon lengths C_16_ to C_22_ (APAA_16–22_), *m/z* 74, 87, 213, 270 for TMTD, *m/z* 74, 88, 101, 312 for pristanic acid, *m/z* 74, 101, 171, 326 for phytanic acid and *m/z* 74, 105, 262, 290, 318, 346 for the detection of ω-(o-alkyl phenyl)alkanoic acids of carbon lengths C_16_ to C_22_ (APAA_16–22_).

### 4.3. Gas chromatography-combustion-isotope ratio mass spectrometry

Stable carbon isotope (*δ*^13^C) values of the major saturated fatty acids (FA; C_16:0_ and C_18:0_) were analysed by GC-C-IRMS. A Delta V Advantage isotope ratio mass spectrometer (Thermo Fisher, Bremen, Germany) linked to a Trace Ultra gas chromatograph (Thermo Fisher) with a GC Isolink II interface was used. This was fitted with a DB-5MS Ultra inert fused silica column (US, 60 m × 0.25 mm × 0.25 µm). One microlitre of sample was injected via a splitless injector maintained at a temperature of 300°C. Helium was used as the carrier gas at a constant flow rate of 3 ml min^−1^. An Agilent 5975C mass spectrometer detector was attached to the column and half of the gas eluting from the column was directed to and ionized in the mass spectrometer. The other half of the gas eluting from the column was directed to the reactor tube to oxidize carbon species in CO_2_. The ionization energy of the mass spectrometer was 70 eV and ion intensities of *m/z* 44, 45 and 46 were recorded.

IonOS software was used to compute the ^13^C/^12^C ratio of the peaks in the extracts. The ^13^C/^12^C ratio was calculated in comparison with a standard reference gas (CO_2_) of known isotopic composition. The delta C values were expressed as per mil (‰) relative to the internal standard V-PDB (Vienna Pee Dee Belemnite). An *n*-alkanoic acid ester standard of known isotopic composition (F8-3) was used to determine the precision and accuracy of the instrument, which needed to remain at less than 0.5‰ and less than 0.3‰, respectively. Values were corrected in relation to the method standard, a mixture of C_16:0_ and C_18:0_ FAs of known isotopic composition.

## 5. Results and discussion

Of the additional 114 samples analysed in this study, a total of 97 samples (85%) yielded appreciable lipids after acidic methylation (electronic supplementary material, S1) above the limit of reliable interpretation (5 µg g^−1^; [33,34]). These results reflect the positive recovery of lipids from samples from Casale San Pietro and Palermo, where greater than 90% of samples yielded appreciable concentrations of lipids [2].

### 5.1. Animal products

**Figure 2.**
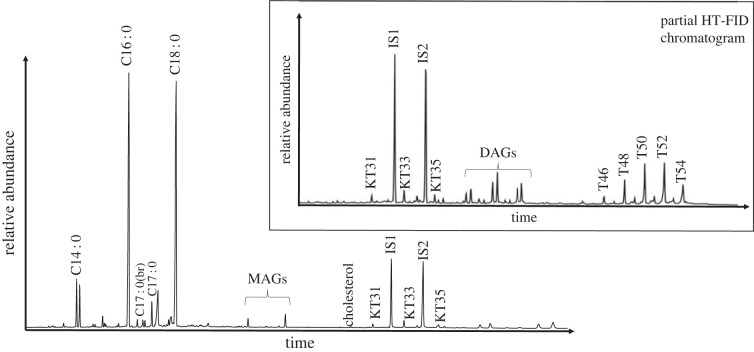
Total ion chromatogram (TIC) and partial HT FID chromatogram of TLE extract of sample MZ_189 typical of ruminant adipose fat. Fatty acids C_14:0,_ C_16:0_, C_18:0_ and branched (br) and unbranched C_17_, cholesterol, ketones (KT), MAGs, DAGs and TAGs (T44–54) are shown alongside the internal standards alkane C_34_ (IS1) and C_36_ (IS2).

Lipid profiles characteristic of degraded animal fats were identified in the majority of all samples analysed. Profiles were dominated by palmitic (C_16:0_) and stearic acid (C_18:0_) in equal proportions. In many cases, branched and linear C_15:0_ and C_17:0_ fatty acids were present, which can be attributed to a ruminant origin, but can also be formed through other bacterial processes [35] (electronic supplementary material, S1). Cholesterol and its derivatives, the major sterol in animal tissues, was also observed in 97 of the total 248 samples. Although cholesterol was not identified alongside squalene (a common skin-lipid contaminant), its presence in archaeological ceramics must be treated with caution as it can derive from handling contamination [36]. Nonetheless, the presence of *n-*ketones C_31_, C_33_ and C_35_ in a number of samples gives clear evidence for the heating of animal fats at temperatures above 300°C [37]. Furthermore, 64 of the samples prepared using conventional SE yielded intact TAGs (electronic supplementary material, S2). Figure 2 shows a typical lipid profile of ruminant adipose fat with TAGs containing between 46 and 54 acyl carbon atoms, with a clear predominance of T_52_ and T_50_ over T_54_ [11]. A broader range of TAGs (T_42_ to T_54_) distinctive of dairy fat origin [11] was observed in two Islamic samples from CLESP and at least two samples from MZ [2] (electronic supplementary material, S2).

### 5.2. Compound-specific isotopic analysis

**Figure 3.**
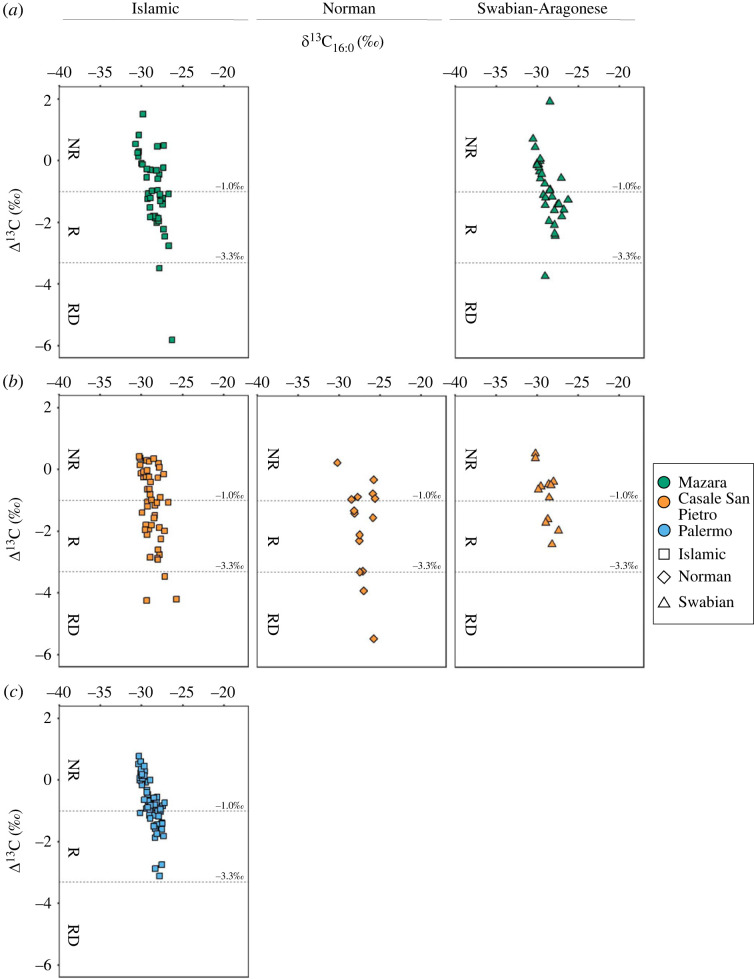
Plots of fatty acid stable isotope values obtained from individual vessels from Sicilian pottery at (*a*) Mazara del Vallo; (*b*) Casale San Pietro; (*c*) Palermo. Plot of Δ^13^C against *δ*^13^C_16:0_. Values less than −3.3‰ are typically associated with RD, values between −3.3‰ and −1.0‰ are associated with R (ruminant adipose) and above −1.0‰ can be considered as NR [38].

Identifying products based on TAG profiles alone can be limited due to the preferential loss of lower molecular weight components [11]. Therefore, to further understand the origin of these lipids, 218/248 of the samples were analysed by GC-C-IRMS to determine the stable carbon isotope value (*δ*^13^C) of the major fatty acids (C_16:0_ and C_18:0_) [2]; figure 3; electronic supplementary material, S1). This analysis helped to distinguish between ruminant adipose, ruminant dairy (RD) fats and non-ruminant fats based on their Δ^13^C (*δ*^13^C_18:0_ − δ^13^C_16:0_) values [11–13]. However, the addition of plant oils to animal fats and/or the sole use of plant products may also influence the Δ^13^C values and the interpretation of the values is generally complicated by mixing [2].

Nevertheless, most of the fatty acid *δ*^13^C values from both Islamic vessels and Swabian vessels from Mazara fall within the range of ruminant adipose (53% and 43%, respectively) and non-ruminant fats (45% and 48%, respectively) (figure 3*a*). Two samples from Islamic contexts and one sample from Swabian contexts at Mazara could be unambiguously attributed to dairy fats (Δ^13^C < −3.3‰). The contribution of dairy fats is also reflected in the broad TAG distribution of two of these samples (MZ_81 and MZ_227; electronic supplementary material, S2). The processing of ruminant adipose is supported by the faunal assemblage at MZ, where caprines dominate in both Islamic and Swabian contexts [19,39]. Cattle, on the other hand, are barely represented at the site, and the faunal assemblage mainly consists of adult and elderly individuals, indicating their use for secondary products such as milk production, manure and agricultural works [39,40].

Many of the Δ^13^C fatty acid values match the range of non-ruminant products, such as poultry or porcine fats or indeed plant oils, and distinguishing these with confidence is problematic. Especially, as none of the samples yielded TAG profiles indicative of porcine or poultry fats. The faunal remains from Mazara show that suid remains were scarce from Islamic contexts, supporting the assumption that pork was rarely consumed as it was prohibited by Islamic hadiths [39,41]. The incidence of suid remains from Mazara increases in the following Swabian and Aragonese phases, but there is no increase in the proportion of ceramics with non-ruminant fats [39,41]. It is possible that, despite religious restrictions, these populations might have consumed wild boar as evidenced in 12th/13th century contexts in Portugal [42]. However, recent works by Aniceti & Albarella [41] indicate that wild boar remains were scarce at these sites and there is little evidence to suggest that hunting of wild animals was a common practice here [41]. Poultry fats perhaps provide the most credible alternative. Certainly, the depleted *δ*^13^C_16:0_ values (approx. −30‰) are within published values from domestic fowl [43]. Other matches include hare [44], but the latter is hardly represented in the Islamic and Swabian faunal assemblage at Mazara [39,40]. By comparing the two periods at MZ, overall, there is no statistical difference between the Δ^13^C values based on a Mann–Whitney U test (W = 633, *p*-value = 0.6763).

The *δ*^13^C values of Norman and Swabian samples from CLESP predominantly fall within the range of both ruminant and non-ruminant adipose fats (figure 3*b*). Previously published *δ*^13^C values from Islamic contexts from CLESP depict an equal distribution of samples falling within the range of ruminant and non-ruminant fats (46% and 48%, respectively) [2]. There is a slight increase in the percentage of samples that fall within the range of non-ruminant fats within the Norman period (34% ruminant compared with 50% non-ruminant values) and again in the Swabian period (41% compared with 58%). However, there is no statistical difference between the distribution of Δ^13^C values across periods, (Kruskal–Wallis test, *x*^2^(2) = 3.74, *p* = 0.15). Furthermore, three Sicilian pots from the Norman period fell within the range of dairy fats, reflecting continued processing of dairy products from the Islamic to Norman periods, whereas no samples from Swabian contexts could be unambiguously attributed to dairy fats.

In comparison with the *δ*^13^C values of samples from Islamic vessels from sites in Palermo, both Mazara and CLESP show a broader range of Δ^13^C values from all chronological periods (figure 3*c*). In Palermo, no samples could be confidently attributed to a dairy origin, marking a clear contrast to contemporary Islamic rural sites [2]. The presence of dairy products in both Islamic and Swabian vessels at the urban centre of Mazara sheds new light on the use of dairy products at urban sites.

### 5.3. Absence of aquatic products

While a small number of samples produced relatively enriched *δ*^13^C_16:0_ fatty acid values (i.e. greater than 27‰) which could indicate the presence of marine products, the majority were more depleted in ^13^C than would be expected for marine products. There was also no observable difference between inland (Casale San Pietro) and coastal locations (Palermo and Mazara del Vallo). An alternative approach is to identify lipid biomarkers for aquatic products, which are well defined [45]. Of these, suites of ω-(*o*-alkyl phenyl) alkanoic acids (APAAs) with 20 and 22 carbon atoms (APAA C_20_ and APAA C_22_), formed through the heating of mono- and poly-unsaturated fatty acids in aquatic oils, are perhaps the most diagnostic. However, these were completely absent in all the samples, despite the use of sensitive methods for their detection. Although the absence of these compounds does not rule out the presence of unheated aquatic oils, as might be expected in fermented fish condiments, it has been shown that APAAs are formed through relatively low-intensity heating and are often observed in cooking pots [46]. In addition, suites of APAAs with 16 and 18 carbon atoms, formed through the heating of terrestrial products (plant and animal), were identified, providing further evidence that many of the pots analysed had been heated (electronic supplementary material, S1).

### 5.4. Plant products

**Figure 4.**
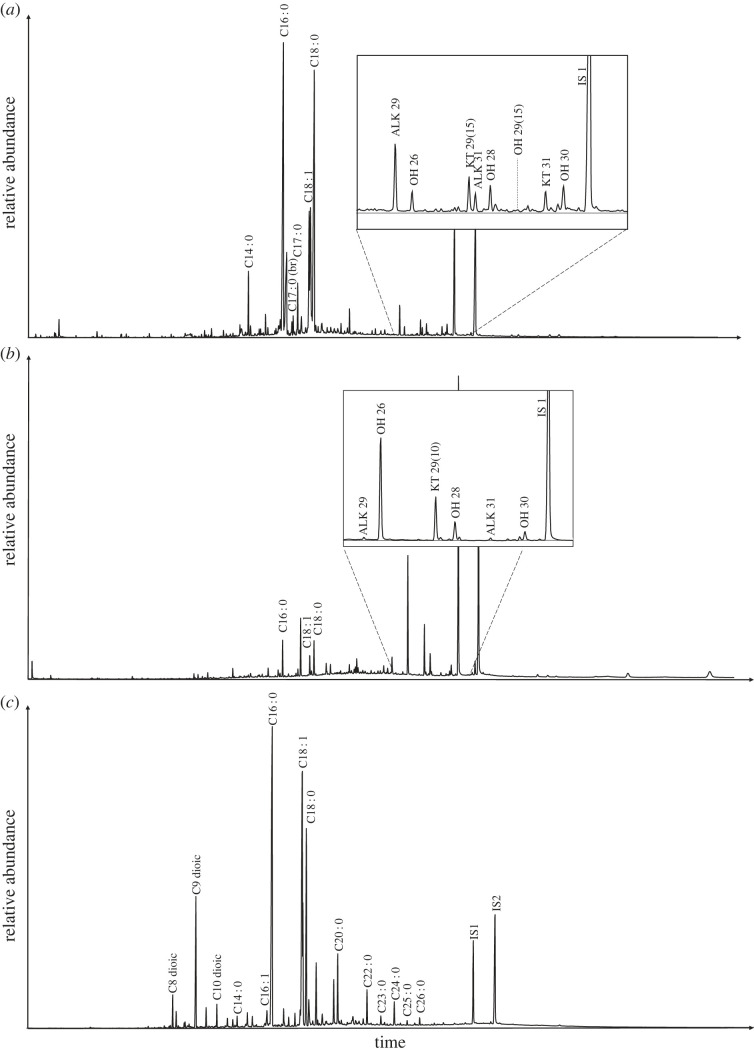
TIC of lipid extracts typical of plant contributions. (*a*) TIC of TLE from sample MZ_14 showing the presence of C_31_ ketone (hentriacontane-16-one) that indicates the presumed presence of leek (*Allium porrum*) in the ceramic samples [37,50–52] and C_29_(15) ketone that indicates the presumed presence of *Brassica* [49]. (*b*) TIC of TLE from sample CLESP_194 showing the presence of C_29_(10) ketone (nonacosane-10-one) that indicates the presumed presence of fennel (*Foeniculum vulgare*) [2,53]. (*c*) TIC of AE from sample CLESP_89 indicating plant oil by a C_18:1_/C_18:0_ > 2 [54,55].

The majority of samples analysed from all sites and periods revealed biomarkers and lipid profiles indicative of a plant contribution (*n* = 77) (electronic supplementary material, S1). The presence of plant sterols (*n* = 22) was frequently observed but does not offer more precise identification because of their ubiquity. Plant waxes were identified in several samples (*n =* 30) by a typical range of *n*-alkanes, *n*-alkanols and *n*-ketones [47,48]. In some cases, these could be attributed to a narrower range of taxa. Nonacosane-15-one and nonacosane-15-ol, in conjunction with a dominance of *n*-alkane C_29,_ can be attributed to *Brassica* [49] and were identified in 20 samples from Islamic and Swabian periods at Mazara, eight samples from Islamic Palermo, but were not observed in any samples from CLESP. Hentriacontane-16-one (C_31_ ketone) and *n*-hentriacontane (C_31_ alkane) attributed to *Allium*, potentially leek [37,50–52], were observed in the same vessels that yielded *Brassica* biomarkers from Mazara (figure 4*a*). However, again, this was not observed in any samples from CLESP.

Furthermore, the ketone nonacosane-10-one previously observed in 10th-12th century ceramics from CLESP and attributed to the possible contribution of fennel (*Foeniculum vulgare;* [2,53] was identified in five further samples from Norman contexts from CLESP (figure 4*b*). Evidence of plant oil, indicated by the relatively high oleic to stearic acid ratio (C_18:1_/C_18:0_ > 2), in addition to a small amount of linoleic acid (C_18:2_; Copley *et al*. [56]; Romanus *et al*. [57]; figure 4*c*) was identified in one vessel from MZ, a jug with soot marks on the side of the vessel, and a further two 12th century samples from CLESP (electronic supplementary material, S1). Plant biomarkers in all sites and periods were often observed in samples that showed evidence of animal fats [2]. Although it is not possible to distinguish between mixing of commodities in a single cooking event and subsequent uses of different products [58], it is highly plausible based on historic accounts that both plants and animal products were mixed in a single recipe [59,60].

Additionally, all solvent extracts were run by GC-MS using a SIM mode to look for fragment ions of alkylresorcinols from cereals (wheat, barley or rye [10]), but these were not observed. However, as they are minor constituents and highly susceptible to degradation, the absence of alkylresorcinols cannot be used to exclude the use of cereals in these vessels [10,54,55].

**Figure 5.**
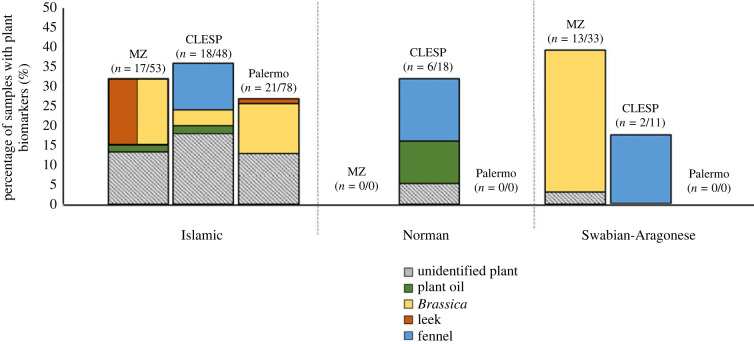
Percentage of ceramic samples from MZ, CLESP and Palermo with plant biomarkers. Unidentified plants are assigned by the presence of sitosterol, stigmasterol and/or specific *n*-alkane distributions. Plant oil is assigned by a C_18:1_/C_18:0_ > 2 [56,57]. C_29_(10) ketone (nonacosane-10-one) indicates the presumed presence of fennel (*Foeniculum vulgare*) [2,53]. C_29_(15) ketone indicates the presence of *Brassica* [49]. C_31_ ketone (hentriacontane-16-one) indicates the presumed presence of leek (*Allium porrum*) [37,50–52]. Actual number of samples out of the total number of samples that yielded plant biomarkers is shown in brackets.

By comparing the presence of plant biomarkers from each of the sites and chronological periods (figure 5), some observations can be made. A wide variety of plant products are observed in the ceramics from each site in the Islamic period, but these varied from one site to another. This diversity seems to persist at CLESP, at least during the Norman period, then disappear during the Swabian period. Conversely, at Mazara, plant consumption seems to focus on brassicas during the Swabian period. While this may indicate less diversity of vegetable products in ceramics as part of post-Islamic cuisines, the difficulties in identifying plant lipids in archaeological ceramics need to be considered, which is hindered by a lack of specific biomarkers and generally lower yields compared with animal products [61].

### 5.5. Fruits and further evidence of plant products

**Figure 6.**
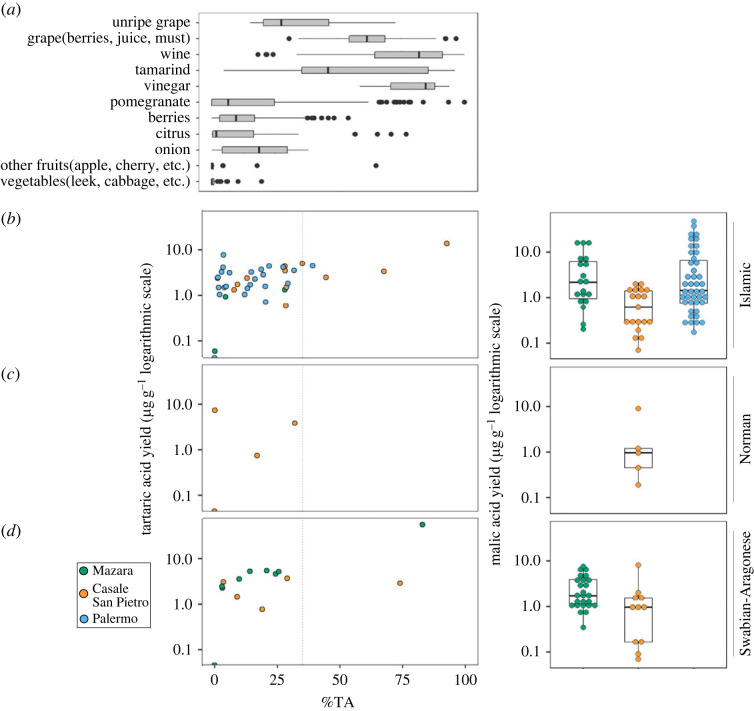
Tartaric yield plotted against %TA and malic acid yield of samples from Mazara, CLESP and Palermo. (*a*) Proportions of tartaric acid in various modern fruits and plant products; (*b*) %TA (tartaric acid/(tartaric + malic acid)) plotted against tartaric acid yield (µg g^−1^) for MZ, CLESP and Palermo across chronological periods and (*c*) Malic acid yield (µg g^−1^) for samples from Mazara del Vallo, Casale San Pietro and Palermo across chronological periods.

Where possible, samples were also extracted using the acid butylation extraction method to investigate the distribution of small organic acids, such as fumaric, succinic, malic and tartaric acid, typically found in fruit products [15,16] (electronic supplementary material, S1). Malic acid and tartaric acid were identified in a number of samples in varying quantities (electronic supplementary material, S1). However, because they are both present in different fruits and plant products [2,14,15], it was important to consider the proportions of the acids individually and in relation to each other to better understand their origin [14] (figure 6). Drieu *et al*. report that the percentage contribution of tartaric acid (%TA) compared with malic acid should be greater than 35% in order to distinguish grapevine products (wine, grape juice and vinegar) from other fruit products (figure 6*a*) [14].

Using this criterion, grapevine products were identified in two ceramic samples; a cooking pot from CLESP and one cooking pot from Mazara, both from Swabian contexts indicating they were used to heat or store wine, or to flavour other foods, as previously suggested in medieval Italy [4,5]. Equally, the results could point to the use of other grapevine products such as vinegar, juices or syrups, which are indistinguishable from wine using this approach. The absence of grapevine markers in Islamic contexts from Mazara mirrors Islamic Palermo and contrasts with its higher occurrence in Islamic pots from the rural site of CLESP [2].

A number of samples yielded greater than 1 µg g^−1^ of malic acid, which is one of the most abundant small acids present in a number of fruits such as plums, apples and peach [62], and therefore may indicate a fruit contribution, other than grapevine, in these vessels. However, it must be considered that malic acid is also present in a variety of other plants including brassicas and leeks and might simply strengthen our identification of these vegetables in the ceramics [63]. Samples that yielded concentrations greater than 1 µg g^−1^ malic acid were more frequently recovered from the urban sites of Mazara and Palermo compared with the rural site of CLESP. These results taken together hint at the more frequent incorporation of fruits in cuisine in urban areas in comparison with rural areas, as suggested in previous research [2]. However, other sources of malic acid need also to be considered.

Importantly, the presence of fruit products in Norman and Swabian samples from CLESP and MZ highlight the continued use of these products in post-Islamic periods. Although it is not possible to identify any variability in the species, these results reflect the archaeobotanical record where, at MZ at least, a continued variety of fruit remains have been identified in Swabian periods [64,65].

### 5.6. Plant resins

**Figure 7.**
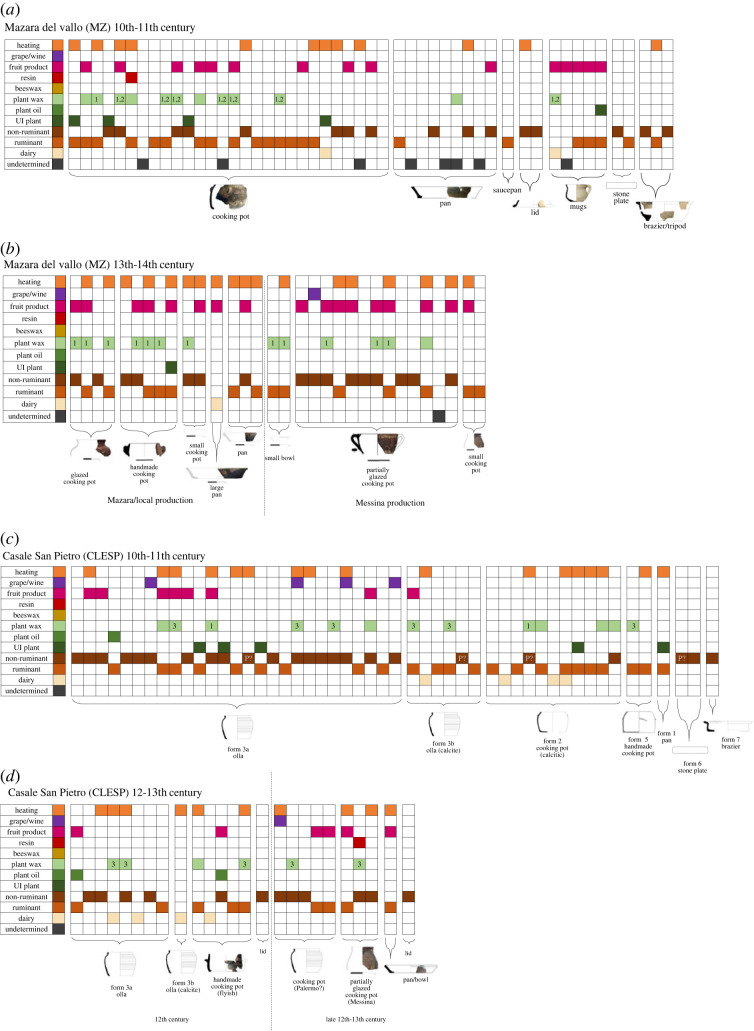
Summary figure of organic substances identified in pottery vessels of specific forms. (*a*) Mazara 10th-11th century ceramics, (*b*) Mazara 13th-14th century ceramics, (*c*) CLESP 10th-11th century ceramics and (*d*) CLESP 12th-13th century ceramics. Identification criteria of different commodities (RD, ruminant adipose, non-ruminant, unidentified (UI) plant, plant oils, plant wax, beeswax, resin (pine/birch-bark), fruit products and grape products) are outlined in the text. Specific taxonomy of plant waxes is indicated: *Brassica* (1), leek (2) and presumed fennel (3).

Overall, plant resins were rarer in the post-Islamic pots from both CLESP and MZ compared with vessels from Islamic contexts [2]; electronic supplementary material, S1, data). The presence of methyl dehydroabietic acid, possibly derived from heated pine resin, was identified in one 13th century sample from CLESP [66,67]. One sample from Mazara (MZ_28) yielded a series of terpenoids indicative of birch-bark tar [68,69], including betulin and lupenol degradation products (lupa 2,20(29)-dien-28-ol and lupa 2,20(29)-diene). Birch-bark tar may have had a range of uses; repairing ceramics, as an adhesive, sealing and as surface decoration. Its presence here may also be attributed to the reuse of this cooking pot for the storage, production or transportation of birch-bark products [70–75]. This sample also yielded evidence of ruminant adipose fats (figure 7*a*) (electronic supplementary material, S3). Fatty products have been shown to modify the consistency or properties of birch-bark tar (e.g. fluidity, mechanical resistance of dry tar etc.) [70–75]. This is the first evidence reported of birch-bark tar used in early medieval contexts outside of Britain and contrasts with the notion that there was a shift from birch tar to pine resin at the beginning of the Roman period [76]. Previously, the most recent attestation of birch pitch in Sicily dates back to the Bronze Age [77]. However, it must be noted that this evidence is limited to only one vessel and without further comparative ORA studies in this area it is not possible to suggest the persistent use of birch-bark tar at this time.

### 5.7. Comparison of vessel use between sites and periods

At all three sites, *ollae* and cooking pots, of all fabrics and forms in all chronological periods (Islamic, Norman, Swabian and Aragonese), frequently show evidence of mixing of animal products, plant products and fruits (figure 7). Alongside molecular markers of heating it is possible to suggest these were general cooking wares, perhaps used to make stews or pottages. As previously suggested, these uses are reflective of Islamic-Arabic cuisines that often involve meat as a base, complemented by vegetables and fruits [2]. Thus, these results indicate a continued use of these general cooking wares in post-Islamic Sicily. It should be noted that it is not possible to confirm whether these mixtures are processed together as part of a single recipe/cooking event or whether this represents the build-up of residues over time over multiple cooking events [58]. Interestingly, despite the introduction of glazed wares in the Swabian period, mostly imported from the east of the island from Messina [26], there does not appear to be a distinct difference in the ways the pots were used. It seems these were simply stylistic changes as opposed to functional changes.

Interestingly, flatter, more open vessel samples from all sites and chronological periods, such as the stone plates and pans, show less mixing and seem to be predominantly used for processing animal products, as only a small number of these types yielded evidence of fruit or plant products. Perhaps, then, these were used for frying meat on its own. Interestingly, one pan sample from Islamic contexts at Mazara and one from Swabian contexts yielded evidence of dairy products, which could have been butter or ghee. The use of dairy in pans contrasts to CLESP, where such products were solely processed in closed forms such as *ollae* and cooking pots (figure 7; [2]).

The braziers from Mazara show evidence of animal fats (figure 7*a*), which probably reflects the dripping of fats and oils when meat was being prepared in a vessel placed above [25]. This is similarly observed in Islamic samples from CLESP and Palermo [2]. Of note, a selection of jugs from Mazara showed evidence of animal fats, plants and fruit products together in the same vessel and may have been used for serving small individual dishes or sauces, and were probably placed directly on the fire resulting in distinctive soot marks observed on many [25]. One 13th century jug from MZ yielded clear evidence of dairy products alongside fruit and plant products, which could reflect a dairy-based sauce or milk that was prepared and flavoured with other products. These results show that the containers had a more complex use than the milk jug hypothesis formulated for North African and Iberian Islamic contexts [78]. A mixture of plant, fruit and potentially milk products has also been observed in 13th century jugs from Piombino, central Italy [4].

In both Islamic and Norman ceramics from CLESP, the majority of pots that contain dairy, sometimes heated, show no evidence of other organic residues and are only found in *ollae* and cooking pots. This suggests that dairy was probably being processed on its own, possibly into cheese, yoghurt or butter in these ceramics as part of the rural economy, as previously observed in vessels from Islamic contexts at CLESP, and supports evidence from the faunal assemblage that a mixed economy prevails at the rural site whereby caprines where readily used for both their meat and milk [2,39]. However, overall evidence for dairy processing in these ceramics is not as frequent as might be expected at this site, and it highly possible that dairy processing took place in other non-surviving containers (i.e. wooden barrels). Interestingly, ceramics with evidence of dairy from Mazara also show evidence of other organic material, such as plant products and fruit products (figure 7). This may represent the multiple uses of these ceramic vessels for different products, but also lends to the idea that these ceramics were being used for recipes containing dairy, and not solely for the processing of dairy products, as observed at CLESP. Furthermore, dairy products at Mazara were present in pans and a jug, but not in *ollae* and cooking pots. The presence of dairy products in ceramic samples from Mazara contrasts with the complete absence of dairy in ceramic samples from the urban sites of Palermo [2]. However, this evidence does indicate that the differential use of dairy products between urban and rural sites remains distinct and is aligned with previous studies which observe similar differences at urban and rural sites in medieval England [79,80].

## 6. Conclusion

This study has investigated changes in culinary practices across the transition from Islamic political control to post-Islamic regimes of the Norman, Swabian and Aragonese periods in Sicily. Overall, there is no evidence of systematic change in the types of foods prepared in pots over this period, despite variability both within and between sites. There is no increase in dairy or reduction in fruit products in the post-Islamic pots as might have been expected if a pastoral economy was reimposed. It is reasonable to assume that Islamic recipes and culinary practices continued following the fall of the Islamic regime and through the late Middle Ages. The continuity in culinary practices supports new dating evidence that shows a persistence of Islamic burial practices in Sicily until at least the 13th century [24]. This also lends substance to the idea that the incoming Normans flourished and profited from their predecessors, and without doubt that they benefited from the agricultural systems, resources and recipes introduced by the Arabs. Thus, highlighting both the continuation of people, ideas and culinary practices as well as the adoption of these habits from incoming people.

However, differences were observed between town and country: for example, the use of dairy products in rural Castronovo as compared with the urban site of Mazara (partial use in recipes) and Palermo (no dairy detected). Other differences were noted in the general use of different wares such as greater mixing of products in closed forms such as *ollae* and cooking pots from all sites and chronological periods in comparison with more restrictive uses of open forms such as pans and stone plates. Only a slight decrease in the variety of plant products processed in ceramic vessels from later periods compared with those from Islamic contexts hints at a change in cuisine.

The application of a multi-faceted organic residue approach to a large number of ceramics was essential here to identify a wide range of products and begin to unravel complex mixtures in the domestic cooking pots. Even so, substantial mixing of animal, plant and fruit products producing a complex palimpsest, potentially formed over many years, hinders and limits our interpretation. It is also important to note that organic residue analyses do not allow for very detailed taxonomic identification, and more nuanced changes may have occurred in the types of ruminants or fruits exploited for example but are undetected. Nevertheless, this study has highlighted the potential of ORA in determining culinary practice in societies subject to changing social and religious pressures. Future refinement of the technique and its broader application promises greater historical insights.

## Data Availability

Additional information pertaining to this article can be found in the electronic supplementary material [81], and all acid extraction GC-MS files can be found in the Dryad Digital Repository: https://doi.org/10.5061/dryad.w3r2280vw [82].
